# Sex-Dependent Metabolic Alterations in Red Blood Cells During COVID-19

**DOI:** 10.3390/biology15050422

**Published:** 2026-03-05

**Authors:** José Raul Herance, Idoia Álvarez-Ajuria, Carolina Aparicio-Gómez, Marina Giralt-Arnaiz, Celia Moya-Latorre, Rita Ortega-Vallbona, Martina Palomino-Schätzlein

**Affiliations:** 1 Molecular Imaging and Therapy Research Group, Vall d’Hebron University Hospital, Autonomous University Barcelona, 08035 Barcelona, Spain; carolina.aparicio@vhir.org; 2 Moldrug AI Systems SL, Parque Tecnológico de Valencia, 46980 Paterna, Spain; ialvarez@moldrug.com; 3 Clinical Biochemistry Research Group, Biochemical Core Facilities, Vall d’Hebron University Hospital, Autonomous University Barcelona, 08035 Barcelona, Spain; marina.giralt@vallhebron.cat; 4 ProtoQSAR SL, Parque Tecnológico de Valencia, 46980 Paterna, Spain; celia070601@gmail.com (C.M.-L.); rortega@protoqsar.com (R.O.-V.)

**Keywords:** red blood cells, COVID-19, NMR, antioxidant metabolism, sex specific alteration, diabetes, cardiovascular diseases

## Abstract

COVID-19 is known to impair red blood cell (RBC) function, which may affect oxygen transport and disease progression. However, the metabolic consequences of SARS-CoV-2 infection on RBCs and how these changes relate to disease severity and sex differences, have not been systematically explored. Here, we compared RBC metabolomic profiles from healthy controls and individuals with COVID-19 using nuclear magnetic resonance (NMR) to gain insight into disease mechanisms and potential biomarkers. Multivariate and univariate analyses were performed within the men and women cohorts, and clinical factors, including body mass index, comorbidities, and critical clinical status were considered. We found that COVID-19 induces significant metabolic remodeling in RBCs of all patients, including reduced glycolytic and pentose phosphate pathway (PPP) intermediates, consistent with impaired energetic and antioxidant capacity. However, we also observed differences between men and women in the pattern and extent of these changes. Female patients showed changes in metabolites implicated in oxygen release and amino sugar metabolism, including 2,3-BPG, suggesting a distinct metabolic adaptation. By contrast, male patients exhibited a broader set of RBC-specific metabolic disruptions, most evident in severe disease, characterized by decreased amino acid levels, altered glycolytic activity, and weakened antioxidant responses. Overall, these findings identify RBC metabolism as a component of COVID-19 pathophysiology and support its potential as a source of biomarkers.

## Simple Summary

Although it is well known that COVID-19 affects red blood cells, the specific metabolic alterations and their relationship to disease outcome have not been fully explored. This study aimed to investigate how COVID-19 alters red blood cell metabolism and whether these changes differ according to sex, disease severity, and major risk factors. Our results show that COVID-19 is associated with significant alterations in red blood cell metabolism, involving pathways related to energy balance, redox homeostasis, and amino acid handling. These changes likely reflect the systemic metabolic stress induced by infection, including hypoxia, inflammation, and oxidative burden. Notably, we observed differences between men and women, with men exhibiting a broader extent of metabolic perturbations, particularly in severe disease. Comorbidities such as diabetes, obesity, and cardiovascular disease further shaped these metabolic patterns, underscoring the integrative role of red blood cells as indicators of systemic pathophysiological states. By highlighting red blood cells as active participants in disease progression rather than passive oxygen carriers, this work identifies a previously underappreciated layer of COVID-19 pathology and underscores the potential of red blood cell metabolism as a source of biomarkers.

## 1. Introduction

While global morbidity and mortality associated with COVID-19 have decreased, SARS-CoV-2 infection remains a serious acute illness for individuals with immunosuppression or other risk factors and continues to be closely monitored [1]. Current variants generally cause similar or milder disease than earlier variants, but more virulent strains could emerge, and population displacement and conflict continue to facilitate spread in settings with limited response capacity [2].

The evolution and outcome of COVID-19 are significantly modulated by different risk factors, including age, sex, obesity, diabetes, and cardiovascular diseases [3,4,5]. Furthermore, around 6% of individuals with symptomatic SARS-CoV-2 infection develop post-COVID-19 condition (PCC), and among these, around 15% continue to experience persistent symptoms after 12 months [6,7]. Recent studies show that, although men have a higher risk for a severe disease outcome, women have a higher risk of developing PCC [8]. In addition, SARS-CoV-2 infection is associated with other long-term sequelae beyond PCC, involving multiple organ systems and contributing to ongoing morbidity and mortality, particularly through increased cardio- and cerebrovascular disease [1].

It has been shown that SARS-CoV-2 infection significantly affects red blood cell (RBC) structure and metabolism, including changes in membrane, lipid composition, and glycolytic metabolites [9,10]. These alterations may be driven by an excessive production of reactive oxygen species (ROS) and systemic oxidative stress reported in COVID-19 [11], leading to elevated levels of oxidized glutathione and decreased oxidative stress defense enzymes, described in RBCs of patients with severe disease [12].

A direct consequence of these alterations could be impaired oxygen delivery, leading to hypoxemia [13,14,15,16]. Comparisons between patients with acute respiratory distress syndrome (ARDS) caused by different diseases have associated an erythrocyte oxidative stress signature specifically with ARDS in COVID-19 patients. In the long term, RBC damage may contribute to PCC, whose underlying mechanisms and full clinical spectrum remain incompletely understood [17]. In this context, a comprehensive study of RBC metabolism could help to investigate the pathogenic mechanisms of COVID-19 in both the short and long term.

Liquid biopsy approaches have been explored as prognostic and diagnostic tools for COVID-19 outcomes and the development of PCC [18,19]. Most strategies focus on detecting viral nucleic acids, although alternative readouts, such as platelet morphology in peripheral blood, have also been proposed [20]. However, the potential of RBC analysis as a liquid biopsy in COVID-19 has not yet been systematically investigated.

Furthermore, many metabolomic studies analyze the metabolic impact of different factors (disease severity, mechanism of action, comorbidities and risk factors, appearance of PCC, virus variants, vaccination status, epidemiological waves) using different biological samples from patients with COVID-19, but not RBCs [21,22,23,24]. A common denominator across these studies has been the identification of amino acids and lipids as the most dysregulated compounds in COVID-19, but organic acids such as citrate, lactate, and 3-hydroxytubyrate, or sugars related to the pentose phosphate pathway (PPP) were also associated with disease severity [25].

Fewer studies have performed analyses stratified by men and women, but available data suggest that COVID-19-associated metabolomic changes are not uniform across groups [26,27,28]. Reported differences indicate more pronounced alterations in men in lipid metabolism and PPP-related metabolites, whereas women more frequently show changes in glycerophosphocholines and carbohydrates. Sex-associated effects have also been reported in aromatic and branched-chain amino acid metabolism, with several studies reporting a greater number of dysregulated metabolites in men.

Metabolomics studies in PCC further suggest that perturbations in lipid and amino acid metabolism, energy metabolism, and mitochondrial function can persist over time, often accompanied by evidence of chronic inflammation [29,30,31,32,33,34].

In this context, characterizing RBC metabolome in COVID-19 could provide mechanistic insight into disease-associated alterations in erythrocyte function and their potential links to clinical course and PCC, thereby complementing previous clinical and metabolomic studies in biofluids. Here, we apply ^1^H nuclear magnetic resonance (NMR) metabolomics to RBCs from patients with moderate and severe COVID-19 and from healthy controls. Given the robustness and high reproducibility of NMR, this approach is well-suited to the highly concentrated erythrocyte extracts obtained from whole blood. To account for reported differences in disease presentation and outcomes between men and women, we conduct analyses separately in male and female cohorts. In addition, we perform stratified analyses in selected subgroups to evaluate the influence of relevant clinical risk factors and disease outcomes on RBC metabolic profiles.

## 2. Materials and Methods

Patients. This study was approved by the Vall d’Hebron University Hospital Ethics Committee for Research with Medicines (protocol PR(AG)335/2020; approval 5 June 2020) and conducted in accordance with the Declaration of Helsinki.

The cohort included 114 adults hospitalized with SARS-CoV-2 pneumonia confirmed by RT-PCR (May–October 2020) and 58 healthy controls with a negative PCR test. Inclusion criteria were age ≥ 18 years and, for patients, RT-PCR confirmation from nasopharyngeal swab and hospital admission. Individuals with comorbidities known to affect RBC physiology (e.g., anemia, sickle cell disease) were excluded.

Subjects were classified as CONTROL, MODERATE, or SEVERE. Severe outcome was defined by ICU admission, acute hypoxemic respiratory failure, acute myocardial injury, acute kidney failure, or death. Demographic data, comorbidities, clinical outcomes, and laboratory variables were collected (key parameters summarized in Supplementary Table S1).

Cohort stratification and subgroup analyses. Analyses were performed separately in men and women. For the primary (“general”) cohort analysis, individuals were selected to achieve comparable distributions of key covariates (age, BMI, and comorbidities) across CONTROL/MODERATE/SEVERE groups; patients with diabetes were excluded from this general cohort. These selections are indicated in Supplementary Table S1, with cohort characteristics summarized in Table S2.

To assess the influence of predefined factors, additional sub-studies were performed for BMI > 30, age > 50, diabetes, and cardiovascular conditions, comparing patients with vs. without each factor, and matching covariates where feasible. For cardiovascular conditions, controls were compared with pooled COVID-19 cases (MODERATE + SEVERE) due to limited sample size. Additional subgroups were defined to evaluate clinical outcome-related variables (oxygenation, ECMO, and death); for ECMO and death, one-to-one matching was used as required by sample size constraints. All subgroup selections are detailed in Supplementary Table S1.

RBCcollection. Whole blood (1 mL) was centrifuged (1600× *g*, 10 min) to isolate the RBC fraction. RBC pellets were stored at 4 °C for up to 72 h and then frozen at −80 °C until extraction.

Metabolite extraction. RBC metabolites were extracted using a methanol/chloroform/water protocol as previously optimized [35]. Frozen RBC pellets stored at −80 °C were thawed on ice for 45 min and extracted with 1.2 mL chloroform (Merck KGaA, Darmstadt, Germany)and 2.4 mL methanol (Merck KGaA, Darmstadt, Germany) (vortexed). Samples underwent two freeze–thaw cycles (submerged in liquid nitrogen for 1 min, then thawed at room temperature). An amount of 1 mL of water was then added, followed by centrifugation at 3000× *g* for 25 min at 4 °C to separate phases. The aqueous phase was collected, solvents were removed by lyophilization, and dried extracts were stored at −80 °C until analysis.

NMR analysis. For NMR, extracts were cooled on ice, reconstituted in 550 μL NMR buffer (100 mM Na_2_HPO_4_ in D_2_O, pH 7.4), and transferred to 5 mm NMR tubes. ^1^H NMR NOESY spectra were acquired on a Bruker AvII 600 MHz spectrometer (Bruker Corporation, Billerica, MA, USA) with presaturation using 256 scans. Spectra were phase- and baseline-corrected and integrated in MestreNova 16 using a predefined integration scheme. Data were normalized to total intensity, excluding solvent and glucose signals [36]. Final metabolite integration values are reported in Supplementary Table S1.

Statistical analysis. Principal component analysis (PCA) was performed in SIMCA-17 to identify outliers and assess clustering. Orthogonal partial least squares-discriminant analysis (OPLS-DA) was used for pairwise comparisons reflecting COVID-19 status and COVID-19 severity (CONTROL vs. COVID-19; MODERATE COVID-19 vs. SEVERE COVID-19). Metabolites with variable importance in projection (VIP) > 1 were retained for follow-up. Multivariate partial least squares (PLS) analysis was performed to assess the correlation between clinical variables and metabolites. OPLS-DA and PLS models were evaluated by cross-validation and permutation testing.

Univariate analyses were performed using custom Python 3.7.11 scripts (SciPy 1.6.3) [37]. Normality was assessed using Shapiro–Wilk, Kolmogorov–Smirnov, and Anderson–Darling tests. Group comparisons used Student’s *t*-test for normally distributed variables and Mann–Whitney U tests otherwise. Multiple testing was controlled using the Benjamini–Hochberg FDR procedure. Boxplots were generated using Python (Matplotlib 3.5.3) [38].

Heatmaps, pathway analysis, and receiver operating characteristic (ROC) analyses were performed in MetaboAnalyst 5.0 [39] using the normalized NMR-derived tables; pathway enrichment *p*-values were adjusted for multiple testing. ROC analysis was conducted in the general cohort (men and women separately) for CONTROL vs. COVID-19 and MODERATE COVID-19 vs. SEVERE COVID-19 comparisons, retaining metabolites with AUC > 0.8 in at least one comparison.

## 3. Results

### 3.1. Altered Metabolic RBC Profile in COVID-19 Patients

**Figure 1:**
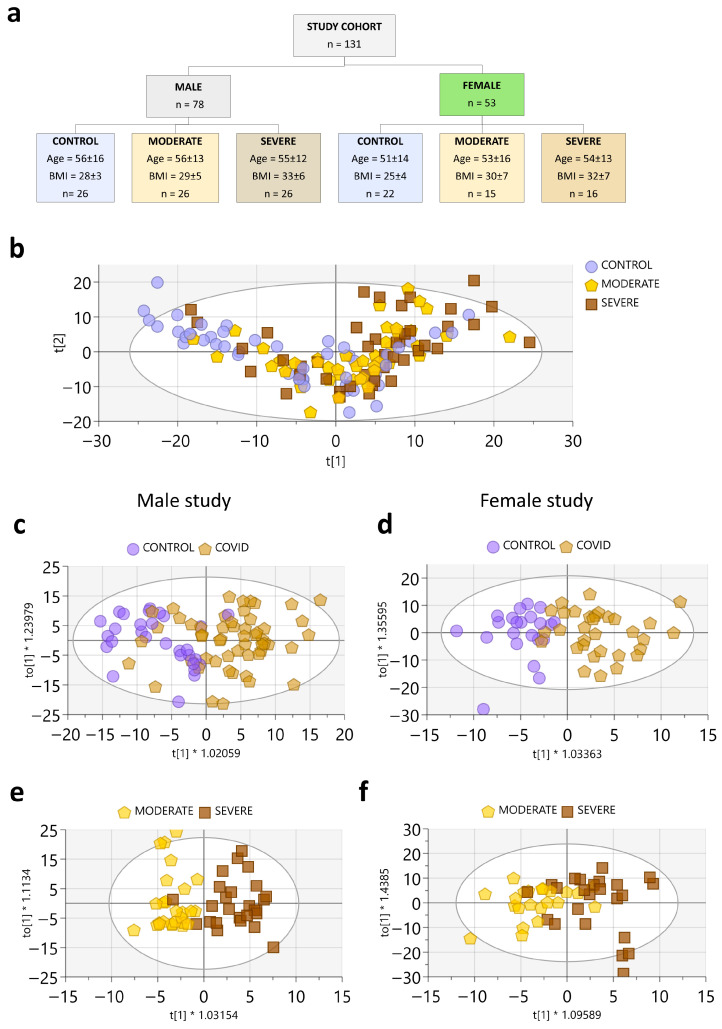
General cohort study. (**a**) Summary of patients included in the general male and female study. Patients were selected to match the main parameters (age, BMI, and comorbidities) across the study groups (control, moderate, and severe). Diabetic patients were not included in this general study. (**b**) PCA score plot of all samples. R2X(cum) = 0.827, Q2(cum) = 0.61. The t[1] and t[2] axes symbolize the principal components 1, and 2, respectively. (**c**) OPLS-DA score plot showing the separate clustering between CONTROL and COVID patient groups in male. R2X(cum) = 0.364, R2Y(cum) = 0.447, Q2(cum) = 0.293. Permutation R2Y = 0.19, Q2 = −0.23. CV-ANOVA = 1.66 × 10^−5^. Data were normalized to total intensity and univariate scaled. (**d**) OPLS-DA score plot showing the separate clustering between CONTROL and COVID patient groups in female. R2X(cum) = 0.426, R2Y(cum) = 0.621, Q2(cum) = 0.299. Permutation R2Y = 0.37, Q2 = −0.49. CV-ANOVA = 0.008. Data were normalized to total intensity and univariate scaled. (**e**) OPLS-DA score plot and S-plot showing the separate clustering between moderate and severe COVID-19 male patient groups. R2X(cum) = 0.348, R2Y(cum) = 0.738, Q2(cum) = 0.224, permutation R2Y = 0.54, Q2 = −0.40, CV-ANOVA = 0.059. (**f**) OPLS-DA score plot and S-plot showing the separate clustering between moderate and severe COVID-19 female patient group. R2X(cum) = 0.348, R2Y(cum) = 0.474, Q2(cum) = −0.294, permutation R2Y = 0.44, Q2 = −0.32, CV-ANOVA = 1. All: R2X(cum) = 0.288, R2Y(cum) = 0.383, Q2(cum) = 0.099, permutation R2Y = 0.383, Q2 = 0.099, CV-ANOVA = 0.074. Data were normalized to total intensity and univariate scaled.

To obtain an overview of COVID-19-related effects on RBC metabolism, we first performed an unsupervised PCA on samples from the general cohort (Materials and Methods; Supplementary Table S2). In the PCA scores plot (PC1 vs. PC2; Figure 1b), the three clinical groups show partial separation: CONTROL samples are more frequent in the upper-left region, MODERATE COVID-19 samples cluster more densely in the lower-central region, and SEVERE COVID-19 samples are more frequent in the upper-right region. This pattern suggests a gradual shift in RBC metabolic profiles with increasing COVID-19 severity, although with overlap between groups.

To define these differences more formally, we performed pairwise OPLS-DA separately in women and men. Models comparing CONTROL vs. COVID-19 (MODERATE+SEVERE) showed clear discrimination in both sexes (Figure 1c,d) and were supported by cross-validation and permutation testing. We then compared MODERATE vs. SEVERE COVID-19. The model in men remained robust and validated (Figure 1e). In contrast, the corresponding model in women showed weaker performance, consistent with a smaller metabolic divergence between MODERATE and SEVERE COVID-19 in the female cohort.

**Figure 2:**
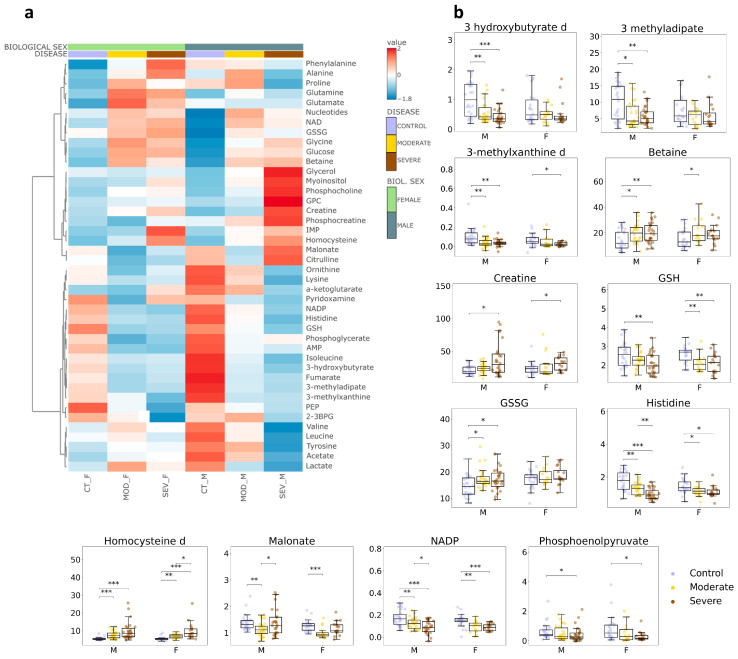
Altered metabolites between CONTROL, MODERATE COVID-19 and SEVERE COVID-19 groups with a similar tendency between male and female. (**a**) Heatmap of significant metabolites in different sample cohorts. CT_F = healthy female individuals, MOD_F = female patients with moderate evolution, SEV_F = female patients with a severe evolution, CT_M = healthy male individuals, MOD_M = male patients with moderate evolution, SEV_M = male patients with a severe evolution. (**b**) Boxplots of altered metabolites between CONTROL, MODERATE, and SEVERE groups with different trends between female and male individuals. GSH = reduced glutathione, GSSG = oxidized glutathione, NADP = Nicotinamide Adenine Dinucleotide Phosphate. * indicates a *p*-value < 0.05, ** indicates a *p*-value < 0.01, *** indicates a *p*-value < 0.001. M = male, F = female.

To identify metabolites associated with COVID-19 status and COVID-19 severity, we next performed univariate testing on metabolites highlighted by the multivariate models (VIP > 1). Results are provided in Supplementary Table S3. An overview of the metabolite patterns across groups is shown in the heatmap (Figure 2a). The heatmap indicates (i) differences between men and women at baseline (CONTROL), (ii) broadly similar COVID-19-associated shifts in both sexes, and (iii) a larger separation between MODERATE and SEVERE COVID-19 in men than in women.

We then examined individual metabolites that showed consistent changes across comparisons. The most representative examples are shown as boxplots (Figure 2b). Across these metabolites, the magnitude of change was generally greater in men.

The metabolites that differentiated the groups included organic acids (3-hydroxybutyrate, 3-methyladipate, malonate), redox-related metabolites (reduced and oxidized glutathione, GSH and GSSG, respectively), energy-related metabolites (phosphoenolpyruvate [PEP], creatine, NADP), and amino acids/derivatives (betaine, histidine, homocysteine).

Several metabolites displayed bigger differences in SEVERE COVID-19, particularly in men. For example, 3-hydroxybutyrate decreased with increasing COVID-19 severity. Malonate showed a non-monotonic pattern, decreasing in MODERATE COVID-19 and partially returning toward CONTROL levels in SEVERE COVID-19.

### 3.2. Metabolites That Change Specifically in Female and Male Patients

**Figure 3:**
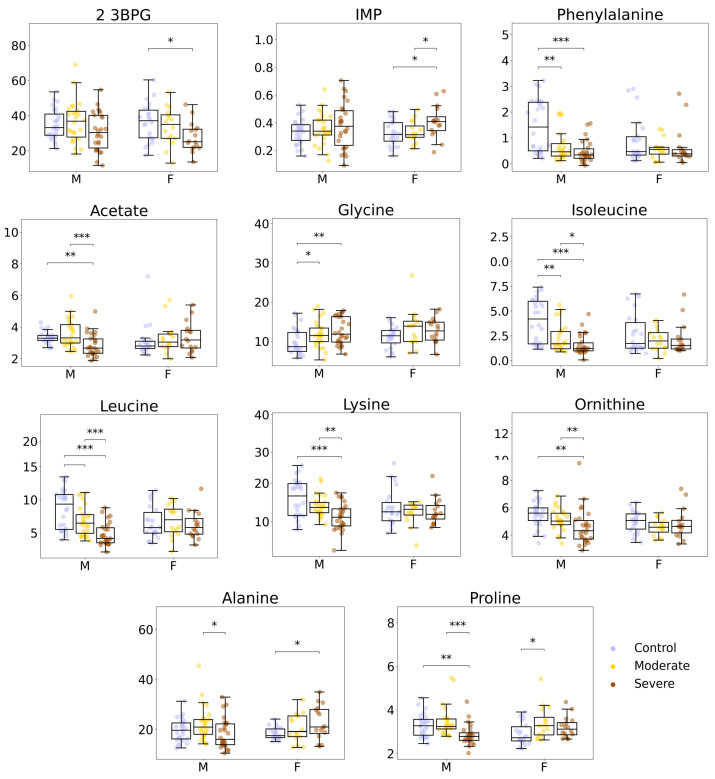
Boxplots of altered metabolites between CONTROL, MODERATE COVID-19 and SEVERE COVID-19 groups with a different trend between female and male individuals. * Indicates a *p*-value < 0.05, ** indicates a *p*-value < 0.01, *** indicates a *p*-value < 0.001. 2,3-BFG = 2,3-bisphosphoglycerate, IMP = inosine monophosphate.

Consistent with the heatmap (Figure 2a), several metabolites showed different COVID-19-associated patterns in men and women. Two metabolites met the significance criteria only in the female cohort: 2,3-bisphosphoglycerate (2,3-BPG) (decreased) and IMP (increased) (Figure 3). Notably, the reduction in 2,3-BPG was primarily observed in women with SEVERE COVID-19.

In men, the amino acids leucine, phenylalanine, isoleucine, ornithine, lysine, and glycine, and the organic acid acetate, were specifically altered (Figure 3). Except for glycine, these differences were more pronounced in SEVERE COVID-19. Most male-associated metabolites decreased, whereas glycine and acetate increased.

Finally, two metabolites showed opposite changes by sex: alanine and proline decreased in men but increased in women (Figure 3).

To further assess differences between men and women, we performed a univariate biomarker analysis using ROC curves. ROC analyses were run separately in male and female cohorts for CONTROL vs. COVID-19 and MODERATE vs. SEVERE COVID-19 comparisons. Metabolites with AUC > 0.8 in at least one comparison are shown in Figure S1.

Several metabolites showed comparable discrimination in both sexes, including homocysteine (increased), NADP (decreased), and histidine (decreased). In contrast, other metabolites showed sex-dependent performance. Myo-inositol (increased) and fumarate (decreased) provided stronger discrimination between CONTROL and COVID-19 in men, and tyrosine (decreased) showed higher discrimination for MODERATE vs. SEVERE COVID-19 in men. Proline displayed a context-dependent pattern: it discriminated CONTROL vs. COVID-19 in women, but MODERATE vs. SEVERE COVID-19 in men, consistent with its opposite direction of change between sexes (decreased in men and increased in women; Figure 3).

No metabolite reached AUC > 0.8 for the MODERATE vs. SEVERE COVID-19 comparison in women, in line with the weaker metabolic separation between these two COVID-19 severity groups observed in the female cohort.

### 3.3. Altered Metabolic Pathways in COVID-19 Patients

After identifying metabolites that differed between groups, we next examined whether these metabolites were over-represented in specific metabolic pathways using pathway enrichment analysis. Enrichment was performed for CONTROL vs. COVID-19 and MODERATE vs. SEVERE COVID-19 comparisons, separately in men and women, using the metabolite lists from the preceding section. Complete pathway enrichment outputs are provided in Supplementary Table S4.

Pathways showing significant enrichment differed by cohort. In both sexes, metabolites mapping to the PPP were enriched. In women, enrichment was also observed for glycolysis-related pathways. In men, enrichment was observed for multiple lipid-related pathways, including de novo triacylglycerol biosynthesis, fatty acid metabolism, sphingolipid metabolism, glycerol phosphate shuttle, glycolipid metabolism, and phospholipid biosynthesis. Additional enriched pathways included amino sugar and aspartate metabolism in women, and glutathione metabolism, sugar metabolism, pyruvate metabolism, and branched-chain amino acid (BCAA) degradation in men.

For the MODERATE vs. SEVERE COVID-19 comparison, pathway enrichment signals were stronger in men than in women. Enriched pathways in men included several amino-acid-related pathways (including arginine, proline, glycine/serine, and BCAA-related pathways) as well as PPP and glycolysis. Because pathway enrichment reflects the over-representation of measured metabolite changes, these results indicate pathways that are statistically associated with the observed metabolite differences rather than direct measurements of pathway activity.

### 3.4. Changes Related to Specific Risk Groups

Because clinical risk factors influence COVID-19 severity, we performed additional subgroup analyses to examine how RBC metabolic profiles varied with age, diabetes, obesity, and cardiovascular (CV) conditions.

**Figure 4:**
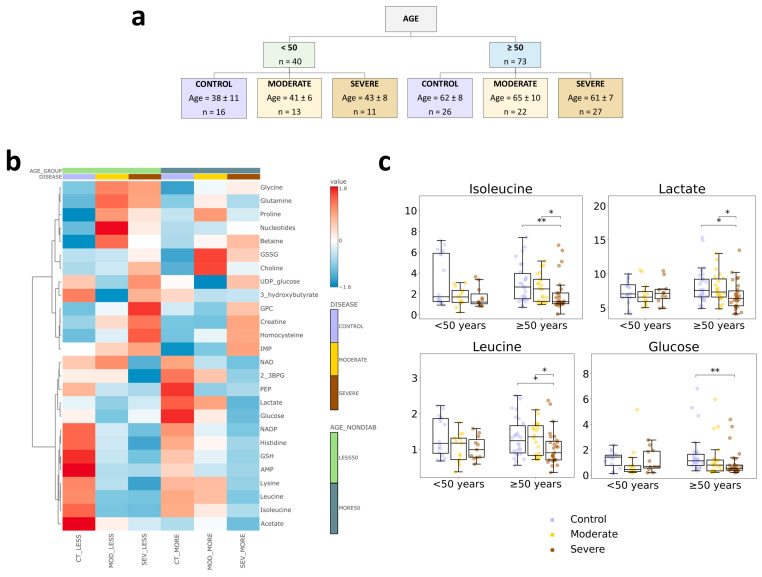
Comparison of CONTROL, MODERATE COVID-19 and SEVERE COVID-19 cohorts of different ages. (**a**) Study design to study the impact of age. Two patient groups were selected: one with age > 50 years and one with age < 50 years. Patients in these groups were matched between the study groups (control, moderate, severe) for the main parameters (sex, age, BMI, and comorbidities). (**b**) Heatmap of significant metabolites in different sample cohorts. CT_LESS = healthy < 50 years individuals, MOD_LESS ≤ 50 years patients with moderate evolution, SEV_LESS ≤ 50 years patients with a severe evolution, CT_MORE = healthy > 50 years individuals, MOD_MORE ≥ 50 years patients with moderate evolution, SEV_MORE ≥ 50 years patients with a severe evolution. (**c**) Boxplot representing the normalized concentrations of metabolites that differ more between <50 years and ≥50 years. * Indicates a *p*-value < 0.05, ** indicates a *p*-value < 0.01.

We first evaluated age by comparing patients < 50 years and ≥50 years, given the association between older age and severe COVID-19 (Figure 4a). The heatmap (Figure 4b) shows broadly similar COVID-19-associated shifts across CONTROL, MODERATE COVID-19, and SEVERE COVID-19 in both age strata, with several age-dependent differences.

In the ≥50 years subgroup, isoleucine, leucine, and lactate showed a larger decrease across COVID-19 groups, consistent with the male-associated patterns identified in the general analysis (Figure 4c). Univariate testing in the ≥50 years subgroup confirmed that these decreases were not observed in older women (Table S5, sheet 2). In addition, glucose differed only in the older subgroup; univariate analysis indicated that this glucose change was present in both men and women (Table S5, sheet 2).

We next evaluated diabetes as a risk factor. Diabetic patients were excluded from the general cohort; therefore, we performed a dedicated analysis comparing diabetic and non-diabetic COVID-19 cohorts. Diabetic and non-diabetic subgroups were selected and matched for sex, age, and BMI (Figure S2a).

The heatmap (Figure S2b) showed that baseline RBC metabolomic profiles differed between diabetic and non-diabetic controls. Despite these baseline differences, many metabolites exhibited similar patterns across CONTROL, MODERATE COVID-19, and SEVERE COVID-19 in both groups, with several changes showing a larger magnitude in the diabetic cohort.

Univariate comparisons indicated that some metabolites significant in the non-diabetic cohort were not significant in the diabetic cohort (Figure S2c; Table S6). Specifically, the glucose decrease observed in the non-diabetic group was not significant in diabetic patients. In addition, changes observed in the non-diabetic cohort but not detected in the diabetic cohort included decreased GSSG, decreased 2,3-BPG (mainly driven by female participants in the non-diabetic cohort), and increased glycine and creatine. By contrast, the decrease in lactate was more pronounced in the diabetic subgroup.

We next assessed the influence of body weight by stratifying participants into those with BMI < 30 and those with BMI ≥ 30 (Figure S3a). This analysis showed baseline differences in RBC metabolic profiles between non-obese and obese healthy controls (Figure S3b). Despite these baseline shifts, COVID-19-associated changes across CONTROL, MODERATE COVID-19, and SEVERE COVID-19 followed broadly similar directions in both BMI strata. However, several differences were larger in the BMI ≥ 30 group (Figure S3c, Table S7). For example, histidine decreased, and sarcosine increased more markedly in obese patients. Lysine, which decreased in male COVID-19 patients in the general analysis, was also more strongly reduced in high-BMI patients, particularly in SEVERE COVID-19. By contrast, quinone decreased specifically in the BMI < 30 subgroup.

Finally, we assessed the potential impact of CV conditions (Supplementary Figure S4a). Due to the limited sample size, MODERATE and SEVERE COVID-19 were pooled in one COVID group (MODERATE+SEVERE). The heatmap (Figure S4b) showed differences between strata (Table S8). In participants with CV conditions, 2,3-BPG did not decrease (Figure S4c), as already happened with the diabetes-stratified analysis; univariate testing indicated that this effect was detected only in women (Table S8, sheet 2). In addition, asparagine decreased in the CV group, a change not observed in the other subgroup comparisons. Conversely, some alterations observed in the general cohort—such as betaine increase and NAD decrease—were not detected in the CV subgroup. Finally, ATP increased in the CV group, whereas it showed a tendency to decrease in the general cohort.

### 3.5. Changes Associated with Clinical Parameters Related to a Severe Outcome

To further complete our study, we also investigated the effects of several clinical parameters that are key determinants of COVID-19 outcomes on alterations in the RBC metabolome. When analyzing the effect of blood oxygen saturation, a critical indicator of severe disease progression, we only identified a limited number of metabolic alterations in this comparison (Figure S5). These included decreases in α-ketoglutarate and alanine, both of which were previously detected as decreased in the general study, α-ketoglutarate predominantly in men and alanine in women (Table S9). In the oxygen-level comparison, the decrease in alanine remained specific to women, whereas the decrease in α-ketoglutarate was observed in both sexes. In addition, glycerophosphocholine (GPC) levels increased with decreasing blood oxygen saturation.

The other two clinical parameters examined were extracorporeal membrane oxygenation (ECMO) and patient death (exitus). Patients receiving ECMO exhibited decreased glycerol levels (Figure S6, Table S10). This metabolite had not been identified as significantly altered in any previous analyses. In addition, choline (previously increased in the general female cohort) and ornithine (previously increased in the general male cohort) were also elevated in ECMO-treated patients. In contrast, GSH, which was decreased in both male and female patients in the general study, was further reduced. In contrast to the general cohort, GSSG was also decreased, a pattern similar to that observed in the obese cohort.

In the comparison of patients with and without exitus (Figure S7; Table S11), increases in two additional metabolites—ascorbate and phosphocreatine—were detected, while lysine, a metabolite previously shown to decrease in male COVID-19 patients, was further reduced.

### 3.6. Correlation Between the RBC Metabolome and Key Clinical Parameters

**Figure 5:**
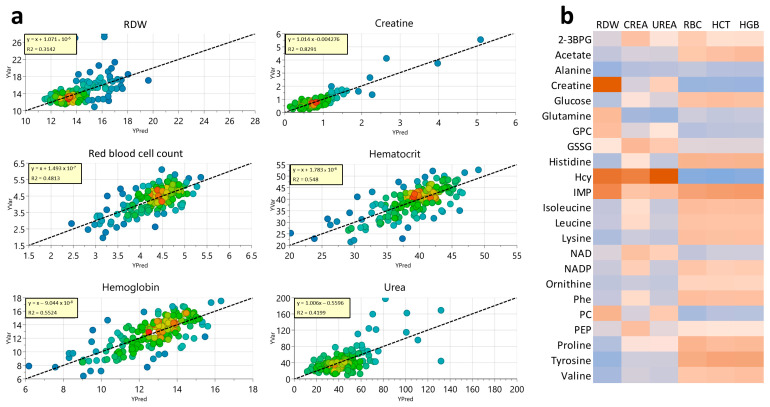
Correlation analysis between metabolomic profiles of RBCs and clinical parameters. (**a**) PLS analysis for the more significative parameters with *p*-value ≤0.01. Dashed lines indicate linear regression plots. (**b**) Heatmap about the intensities of the metabolites in the parameters. Colored in red are the ones with the highest intensity, those colored in orange have a medium intensity and those colored in blue, with lower levels. CREA = blood plasma creatine levels, UREA = blood plasma urea levels, RDW = red blood cell distribution width, RBC = red blood cell count, HCT = hematocrit, HGB = hemoglobine, 2-3BPG = 2,3-bisphosphoglycerate, GPC = glycerophosphocholine, Hcy = homocysteine, IMP = inosine monophosphate, NAD = nicotinamide adenine dinucleotide, NADP = nicotinamide adenine dinucleotide phosphate, Phe = phenylalanine, PC = phosphocholine, PEP = phosphoenolpyruvate.

Finally, we also performed a correlation analysis between the RBC metabolomic profile and various clinical parameters using discriminant PLS. Significant correlations were observed for red cell distribution width (RDW), plasma creatine and urea levels, number of RBCs, hematocrit (HCT), and hemoglobin (HGB) (Figure 5a).

Figure 5b shows a heatmap with the most significant metabolites for each correlation. It can be seen that the metabolic correlations with the parameters RBC, HCT, and HGB are quite similar. This is not unexpected, as the three parameters are closely related. Interestingly, the correlation with RDW seemed to be inverse to this correlation for many metabolites.

Notably, most of the significant metabolites associated with these correlations have been identified as relevant to COVID-19, with changes that are more pronounced in severe disease. Interestingly, many metabolites that increase with the amount of RBC, HCT, and HGB levels decrease in patients with severe or moderate COVID-19, such as acetate, histidine, and BCAA. In contrast, those that increase in COVID-19 patients (alanine, creatine, homocysteine, etc.) correlate negatively with RBC, HCT, and HGB parameters.

## 4. Discussion

In this study, NMR metabolomics were used to reveal the impact that SARS-CoV-2 infection has on RBC metabolism. Unsupervised and supervised multivariate analyses showed a shift in RBC metabolic profiles from healthy controls to COVID-19 cases, which is more pronounced in SEVERE COVID-19 men. In addition, stratified analyses indicated that several patient factors modulate these RBC metabolic patterns and should be considered when interpreting disease mechanisms and evaluating potential biomarkers.

### 4.1. Core COVID-19 RBC Signature: Redox and Energy Metabolism

Across the cohort, COVID-19 was associated with a reproducible set of RBC metabolic changes observed in both men and women and largely preserved across risk-factor stratifications, as summarized in Figure S8. Thus, we observed coordinated changes in metabolites related to redox balance and energy metabolism, including reduced GSH, pyridoxamine, NADP/NADPH, phosphoenolpyruvate, and AMP, together with decrements in additional metabolites (such as 3-hydroxybutyrate, citrulline, fumarate, malonate, and phosphocholine). In parallel, homocysteine, IMP, and myo-inositol increased. These shared changes are consistent with the oxidative and metabolic stress associated with COVID-19.

A central finding was the alteration of the glutathione/redox system. Decreases in GSH and pyridoxamine, together with changes in NADP/NADPH, suggest a disruption of RBC reducing capacity under COVID-19-associated oxidative stress [40,41]. Because RBCs rely on the PPP as their main source of NADPH, these results support involvement of pathways that maintain antioxidant defenses. However, metabolomics does not directly measure pathway flux. In addition, reduced phosphoenolpyruvate and AMP may indicate perturbations in glycolytic/adenylate-related metabolism, consistent with a shift in RBC energy–redox balance during [42,43].

Some metabolic changes may correlate with alterations in membrane-related metabolism and altered RBC deformability, such as the increase in myo-inositol and decrease in phosphocholine; myo-inositol has been implicated in RBC membrane homeostasis and showed strong discrimination in men in our ROC analysis (AUC > 0.8; Figure S1) [44].

Overall, COVID-19 is associated with a consistent pattern of RBC metabolic alterations reflecting systemic oxidative and metabolic stress, with additional changes emerging in severe disease.

### 4.2. More Pronounced RBC Metabolomic Remodeling in Men with Severe COVID-19

Beyond the shared COVID-19-associated RBC metabolic changes, our sex-stratified analyses showed differences in both the pattern and size of metabolite changes between women and men (Figure S8). In women, significant changes not seen in men included lower 2,3-BPG and UDP-glucose and higher choline and proline. Because 2,3-BPG regulates hemoglobin oxygen affinity, this finding may be relevant to oxygen-related RBC physiology during infection [45]. The 2,3-BPG decrease was mainly observed in women with severe COVID-19. It was not detected in women with diabetes or cardiovascular comorbidities, suggesting that these factors can modify or mask this association in subgroup analyses. Differences in UDP-glucose were consistent with pathway enrichment pointing to amino-sugar-related pathways in women [46].

In men, we observed greater metabolite differences and a clearer separation between moderate and severe COVID-19 in multivariate models, as most changes were significantly more pronounced in the severe state of the disease (Figure 1 and Figure 3). These male-associated changes included lower levels of several amino acids (phenylalanine, isoleucine, leucine, valine, lysine, ornithine, tyrosine) and organic acids (α-ketoglutarate, lactate, acetate). Similar classes of amino-acid alterations have been reported in COVID-19, and RBC measurements may partly reflect broader changes in amino acid availability and exchange in blood [47]. Several amino acids also showed higher discrimination in men in the ROC analysis (Figure S1). BCAA changes were most evident in men ≥ 50 years, suggesting that age modulates these patterns [48,49]. Ornithine was also altered in ECMO-treated patients, indicating overlap with severity-related subgroups [50].

Alanine changed in opposite directions by sex (decreased in men, increased in women), which may reflect differences in metabolic context rather than a single mechanism [51]. Redox-related metabolites also differed by sex (e.g., α-ketoglutarate and glycine decreased in men, while proline showed opposite directions) [52].

Overall, these findings indicate sex- and comorbidity-associated differences in RBC metabolite patterns in this cohort. The broader spectrum of amino acid and organic acid alterations observed in men may suggest a more pronounced catabolic and metabolically stressed state, consistent with the greater severity of COVID-19 reported in this sex. In women, a less adaptive reaction may contribute to chronic microcirculatory and metabolic dysfunction, related to PCC. However, replication and complementary functional measurements are required to confirm this hypothesis.

### 4.3. Risk Factors and Comorbidities as Modifiers of RBC Metabolic Patterns

Several RBC metabolite changes associated with COVID-19 differed across risk-factor and comorbidity subgroups, indicating that baseline patient characteristics can influence the detectability and magnitude of these associations (Figure S8). In addition to the examples discussed above (such as 2,3-BPG and BCAAs), this effect modification was also observed for creatine. Creatine levels increased in COVID-19, particularly in severe disease, but this increase was not detected in the diabetic subgroup. In our cohort, RBC creatine also showed a positive correlation with RDW (Figure 5). Elevated RBC creatine has been reported in settings of cardiopulmonary insufficiency [53], and lower baseline circulating creatine has been described in diabetes [54], which may contribute to differences observed across subgroups.

A second example is GSSG. GSH/GSSG balance was altered in COVID-19 overall, with an increasing GSSG tendency, especially in men. However, this increase was not detected in diabetic patients, and GSSG decreased in obesity and in ECMO-treated patients. These subgroup differences are consistent with the possibility that pre-existing oxidative stress or altered redox homeostasis in these conditions may influence the glutathione response observed during SARS-CoV-2 infection [55].

Cardiovascular comorbidities also modified several associations, including 2,3-BPG, acetate, betaine, and NAD. In addition, asparagine and ATP showed changes specifically in the subgroup with both COVID-19 and cardiovascular disease. These findings suggest that comorbidity-related baseline differences and disease interactions can shape the RBC metabolic profile and should be considered when comparing cohorts and when proposing biomarkers [56].

Finally, in the subgroup analyses related to fatal outcome, ascorbate, lysine, and phosphocreatine differed between survivors and non-survivors. However, these associations should be interpreted cautiously, given the limited size of outcome-defined subgroups, but they motivate further evaluation in larger cohorts [57].

### 4.4. Limitations and Future Directions

These results support the potential of RBC metabolomics to capture clinically relevant variation in COVID-19, as reflected by the ROC analyses and the multivariate associations with selected clinical variables. However, the biomarker candidates highlighted here should be considered exploratory until confirmed by targeted quantitative assays and validated in independent cohorts. Several limitations should also be acknowledged: this study was performed in a single, single-center cohort; participants were recruited during early pandemic waves (unvaccinated, first infection), and metabolomic profiles may differ with vaccination, reinfections, and viral variants; and metabolomics identifies statistical associations but does not directly measure pathway flux or RBC function. Accordingly, longitudinal and multicenter studies, ideally combined with functional RBC measurements (such as deformability, oxygen affinity, oxidative damage markers), are needed to assess generalizability and clarify physiological relevance. Because several associations were influenced by sex and comorbidities, future work should incorporate stratified analyses and/or formal interaction testing and evaluate whether multi-metabolite panels improve performance over single markers.

## 5. Conclusions

This study demonstrates that COVID-19 induces significant metabolic alterations in RBCs, affecting key pathways related to energy metabolism, redox balance, amino acid transport, and oxygen delivery. Several metabolic changes were observed independently of sex or risk factors, including decreases in glycolytic and PPP intermediates, suggesting a general impairment of RBC energy and antioxidant metabolism during infection. These alterations may contribute to oxidative stress, reduced RBC deformability, and impaired oxygen transport.

Importantly, our data reveal several sex-associated metabolic responses. Female patients showed distinct alterations in metabolites related to oxygen release and amino acid metabolism, such as 2,3-BPG, which may reflect differences in metabolic adaptation and could be relevant to both acute disease severity and PCC, although this requires confirmation in longitudinal studies.

In contrast, male patients exhibited a higher number of RBC-specific metabolic disturbances, including reduced amino acids, altered glycolytic flux, and impaired antioxidant responses, particularly in severe cases. These findings suggest a greater systemic metabolic impact of COVID-19 in men.

Furthermore, the presence of comorbidities such as diabetes, obesity, and cardiovascular disease modulated several metabolic responses. This indicates that pre-existing metabolic stress may affect the response of RBCs upon SARS-CoV-2-induced oxidative and inflammatory challenges. Overall, RBC metabolomics emerges as a valuable tool to capture systemic metabolic dysregulation, sex-specific disease mechanisms, and the influence of comorbidities in COVID-19.

## Data Availability

All data of the study are available in the supporting material or upon demand.

## Supplementary Materials

The following supporting information can be downloaded at: https://www.mdpi.com/article/10.3390/biology15050422/s1, main clinical data of all patients, groups selection and normalized integration data can be found in Supporting Table S1. Summary of main parameters of male and female patients of the general cohort study are reflected in Table S2. Table S3 provides the results of pathway enrichment analysis. Selected ROC curves from biomarker analysis are reflected on Figure S1. Data and additional figures for the study of general (Table S3), age (Table S5), diabetic (Table S6, Figure S2), BMI (Table S7, Figure S3), CV (Table S8, Figure S4), oxygen saturation (Table S9, Figure S5), ECMO (Table S10, Figure S6) and exitus (Table S11, Figure S7) are available. Table S4 provides a summary of the pathway enrichment analysis. Figure S8 provides a summary of the main metabolic findings of the articles.
