# Supplementary material for: Sex-Dependent Metabolic Alterations in Red Blood Cells During COVID-19
Source: Biology (Basel). 2026 Mar 5;15(5):422. doi: 10.3390/biology15050422 (PMC12985284; doi:10.3390/biology15050422)
Supplement: Supplementary file 1 [file biology-15-00422-s001.zip › Supplementary Table S2.pdf]

Table S2. Main clinical and biometric parameters of patients of the male and female general cohort study.

	MALE					FEMALE				
**Variable**	**CONTROL**	**MODERATE**	**SEVERE**	**p CT/MOD**	**p CT/SEV**	**CONTROL**	**MODERATE**	**SEVERE**	**p CT/MOD**	**p CT/SEV**
**n**	**26**	**26**	**26**			**22**	**15**	**16**		
exitus	0/26	1/25	9/15			0/22	1/14	4/12		
ECMO	0/26	0/26	7/19			0/22	0/15	3/13		
O2 saturation (%)	N/A	1±0	93.9±4.1			N/A	94.9±2.5	96.8±1.8		
BMI	27.3±3.2	29.1±5	33.9±5.8	0.319	0.017	25.3±4	30.9±6.7	33.2±6.9	0.018	0.002
Age (years)	55.2±15.5	55.7±12.8	55.6±11.5	0.915	0.928	51.3±13.9	56.5±14.1	53.4±12.4	0.278	0.641
Hematocrit	45.5±4.1	41.4±2.9	32.2±6.2	0.000	0.000	40.6±3.4	40±3.8	32.7±4.4	0.293	0.000
RDW	13.2±0.9	12.9±1.4	15.5±2.2	0.107	0.000	13.1±1	13±0.9	14.8±2.4	0.852	0.016
Neutrophils (c/ml)	4.4±2.1	5.3±2.7	7.9±3.8	0.122	0.000	4.4±2.4	5.1±3.1	8.5±4.3	0.338	0.001
MCHC (g/dl)	33.4±1.3	33.3±1	31.7±1.5	0.447	0.000	32.7±1	32.2±1.3	31.7±1.4	0.278	0.010
Eosinophils (%)	2.3±1.7	0.6±1	4.2±6.4	0.000	0.216	3.4±5.5	0.4±0.7	0.8±1.3	0.000	0.000
Monocyts (%)	7.9±2	7.5±3.1	5.2±2.3	0.431	0.000	8±1.8	7.3±1.4	5.7±2.4	0.220	0.002
Serum LDH (U/L)	N/A*	339.6±95.4	452.8±184.6			N/A*	282.2±52.8	349.4±115.3		
PT (s)	100.5±21.8	94.6±14.4	81.4±20.8	0.070	0.002	109.6±9.4	97.6±15.2	96.7±14.3	0.032	0.002

ECMO = extracorporeal membrane oxygenation, BMI = body mass index, LDH = lactate dehydrogenase, hematocrit = % RBC in blood, RDW = Red Cell Blood Distribution Width, MCHC = Mean Corpuscular Hemoglobin Concentration, c/ml = cels per ml, PT = Prothrombin Time. *Standard values of LDH in blood serum are 140–300 U/L.
