# Supplementary material for: Sex-Dependent Metabolic Alterations in Red Blood Cells During COVID-19
Source: Biology (Basel). 2026 Mar 5;15(5):422. doi: 10.3390/biology15050422 (PMC12985284; doi:10.3390/biology15050422)
Supplement: Supplementary file 1 [file biology-15-00422-s001.zip › Supplementary Table S4.pdf]

Table. S4 Metabolic routes related to altered metabolites in RBCs. Differences between sex and between control-covid and moderate-severe patient groups.

		CONTROL - COVID				MODERATE - SEVERE			
		Female		Male		Female		Male	
Pathway	Tot Comp	Hits	P-value	Hits	P-value	Hits	P-value	Hits	P-value
**Alanine Metab**	17	2	*	2	***				
**Amino Sugar Metab**	33	3	**						
Ammonia Recycling	31	5	**	5	*				
Androgen and Estrogen Metab	33	2	+	2	**				
Androstenedione Metab	24	2	+	2	***				
Arachidonic Acid Metab	67			4	**				
**Arg and Proline Metab**	52			8	*			8	*
**Aspartate Metab**	35	5	**						
Beta-Alanine Metab	34			4	*				
Betaine Metab	21	3	*	3	***				
Bile Acid BioSyn	65	3	*	3	**				
Biotin Metab	8			1	***			1	**
Butyrate Metab	19			1	**				
Caffeine Metab	23			1	**				
Cardiolipin BioSyn	11			1	**				
Carnitine Synthesis	22	3	+	3	***			3	*
Catecholamine BioSyn	20	1	**	1	***	1	*	1	*
Citric Acid Cycle	32			1	**				
**Cysteine Metab**	26			2	+				
**De Novo Triacylglycerol BioSyn**	9			1	**				
**Deg of Superoxides**	11	1	***	1	***			1	*
Estrone Metab	24	2	+	2	**				
**Fatty Acid BioSyn**	35	2	*	3	+				
**Fatty acid Metab**	43			1	**				
**Folate Metab**	29			3	+				
**Fructose and Mannose Deg**	31	2	+	2	**				
**Galactose Metab**	38			1	**				
Glucose-Alanine Cycle	13			3	+				
Gluconeogenesis	33	4	*			4	+	4	+
**Glutamate Metab**	48	7	**	7	**				
**Glutathione Metab**	20	5	+	5	***				
Glycerol Phosphate Shuttle	11			1	**				
**Glycerolipid Metab**	25			2	**				

**Glycine and Serine Metab**	59	7	*	7	**			7	*
**Glycolysis**	23	3	*			3	+	3	+
Histidine Metab	42			4	*				
Homocysteine Deg	9	1	**	1	***	1	*	1	*
Inositol Metab	30			1	**				
Inositol Phosphate Metab	24			1	**				
Ketone Body Metab	13	2	+	2	***				
Lysine Deg	30			4	***			4	**
Malate-Aspartate Shuttle	10			2	+				
**Methionine Metab**	42	4	**	4	***				
Methylhistidine Metab	4	1	**	1	***			1	**
Nic and NAM Metab	35	4	**						
**Nucleotide Sugars Metab**	20			1	**				
**Pentose Phosphate Pathway**	29	1	***	1	***			1	*
Phenylacetate Metab	9	1	**						
Phe and Tyrosine Metab	20			2	*				
**Phosphatidylcholine BioSyn**	14	1	*	1	**			1	*
**Phospholipid BioSyn**	29	2	*	2	**			2	**
Phytanic Acid Peroxisomal Ox	26	2	+	2	**				
Plasmalogen Synthesis	26	2	+	2	**				
**Porphyrin Metab**	40	2	*	2	**				
Propanoate Metab	42			2	+				
Pterine BioSyn	28	2	+	2	**				
**Purine Metab**	73	6	**	6	*				
**Pyrimidine Metab**	57	2	**						
Pyruvaldehyde Deg	10	1	***	1	**				
**Pyruvate Metab**	47			6	*			6	+
Retinol Metab	35			3	*			3	**
**Selenoamino Acid Metab**	27			1	**				
**Spd and Spm BioSyn**	18			1	*			1	+
Sphingolipid Metab	40	2	*	2	**			2	**
**Starch and Sucrose Metab**	31			1	**				
Steroid BioSyn	48	1	***	1	***			1	*
Steroidogenesis	43	2	+	2	**				
Thr and 2-Oxobutanoate Deg	20			1	**				
Tryptophan Metab	59			3	+				
Tyrosine Metab	70			3	+				
Ubiquinone BioSyn	20	1	***	1	***			1	*

Urea Cycle	28	5	**						
**Val, Leu and Ile Deg**	59			4	***			4	**
Vitamin K Metab	13	1	***	1	***			1	*
Warburg Effect	57	6	**	6	+				

(For a clearer vision, the p-values have been replaced by the following signs: > 0.1 = +, 0.05-0.01 = *, 0.01-0.001 = **, < 0.001 = ***). Metab = metabolism, BioSyn = biosynthesis, Deg = degradation, Ox = oxidation, Tot Comp = total number of compounds in the pathway, Hits = number of altered compounds in the pathway. Pathways that take place directly in RBCs are labelled in bold.
