# Supplementary material for: Sex-Dependent Metabolic Alterations in Red Blood Cells During COVID-19
Source: Biology (Basel). 2026 Mar 5;15(5):422. doi: 10.3390/biology15050422 (PMC12985284; doi:10.3390/biology15050422)
Supplement: Supplementary file 1 [file biology-15-00422-s001.zip › Supplementary_figures.pdf]

Supplementary Figures

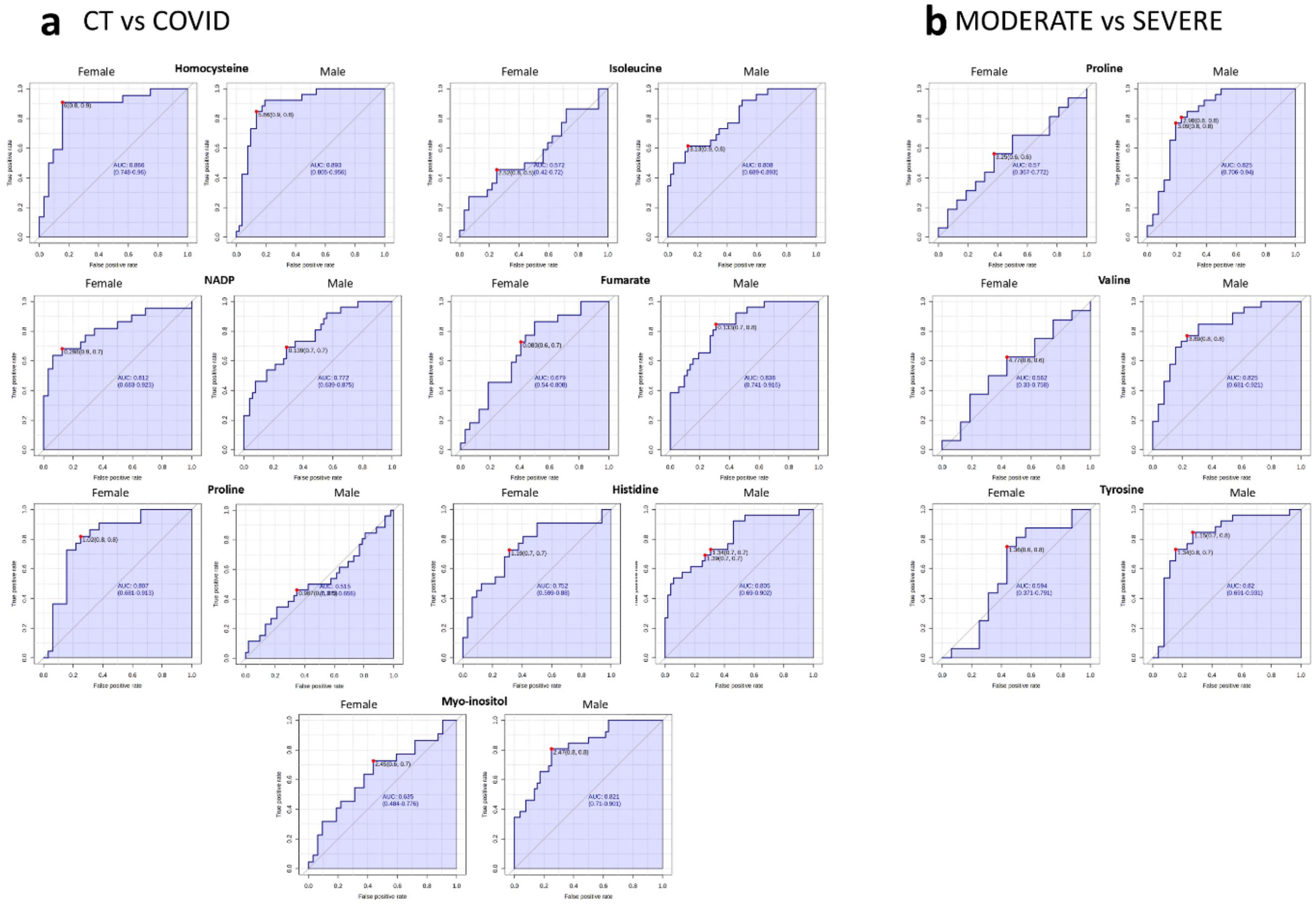

Figure S1. ROC curve analysis. ROC curves of metabolites with an AUC (area under the curve) > 0.8 of the comparisons CONTROL versus COVID (a and b), and MODERATE versus SEVERE (c), separately for male and female subjects. No metabolites with AUC > 0.8 for the comparison MODERATE versus SEVERE were identified for female subjects.

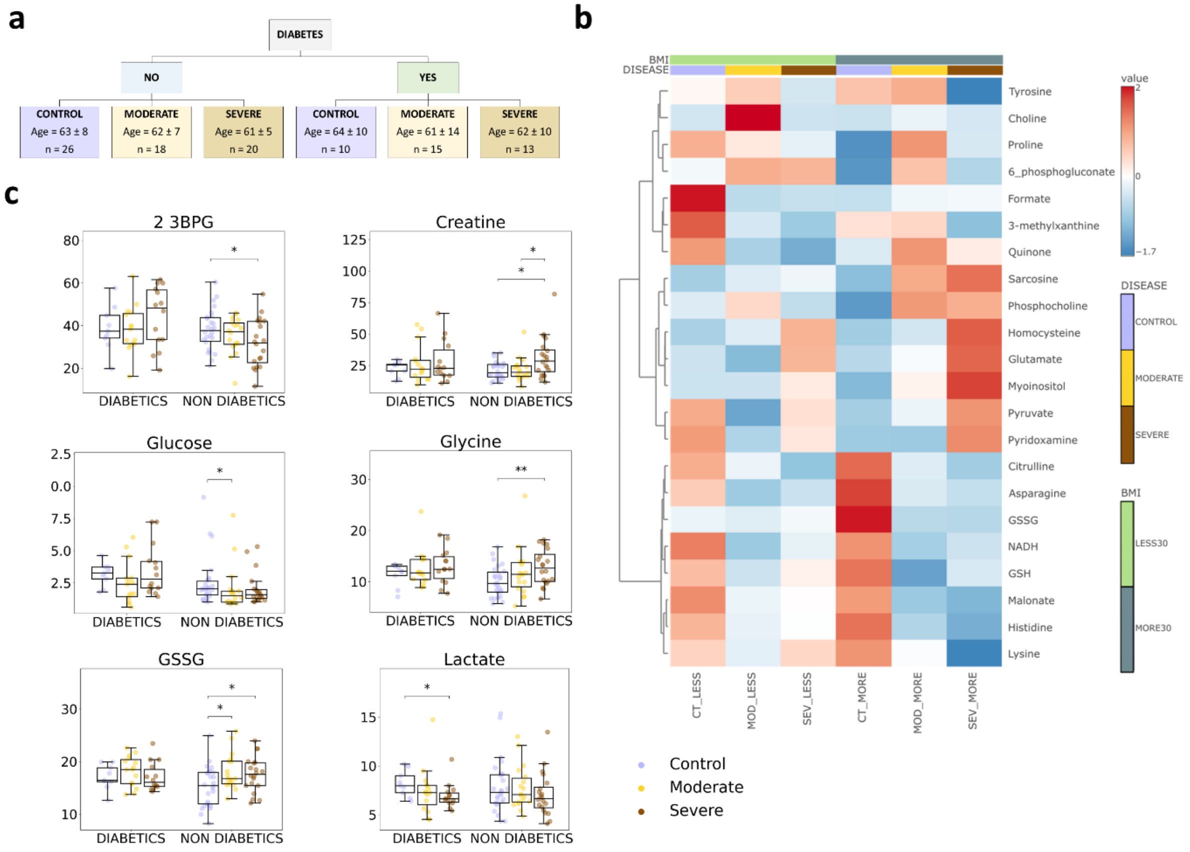

Figure S2. Specific changes in Diabetes. A) Diabetes separation into diabetics and no diabetics and further division into the three different variables: CONTROL, MODERATE COVID-19 and SEVERE COVID-19. B) Heatmap. C) Boxplot representing the normalized concentrations of metabolites that differ between diabetics and non-diabetics. * indicates a p-value < 0.05, ** indicates a p-value < 0.01. PEP = phosphoenolpyruvate, NAD = Nicotinamide adenine dinucleotide, GSSG = oxidized glutathione, AMP = adenosine monophosphate, 2 3BPG =2,3-bisphospyhoglycerate.

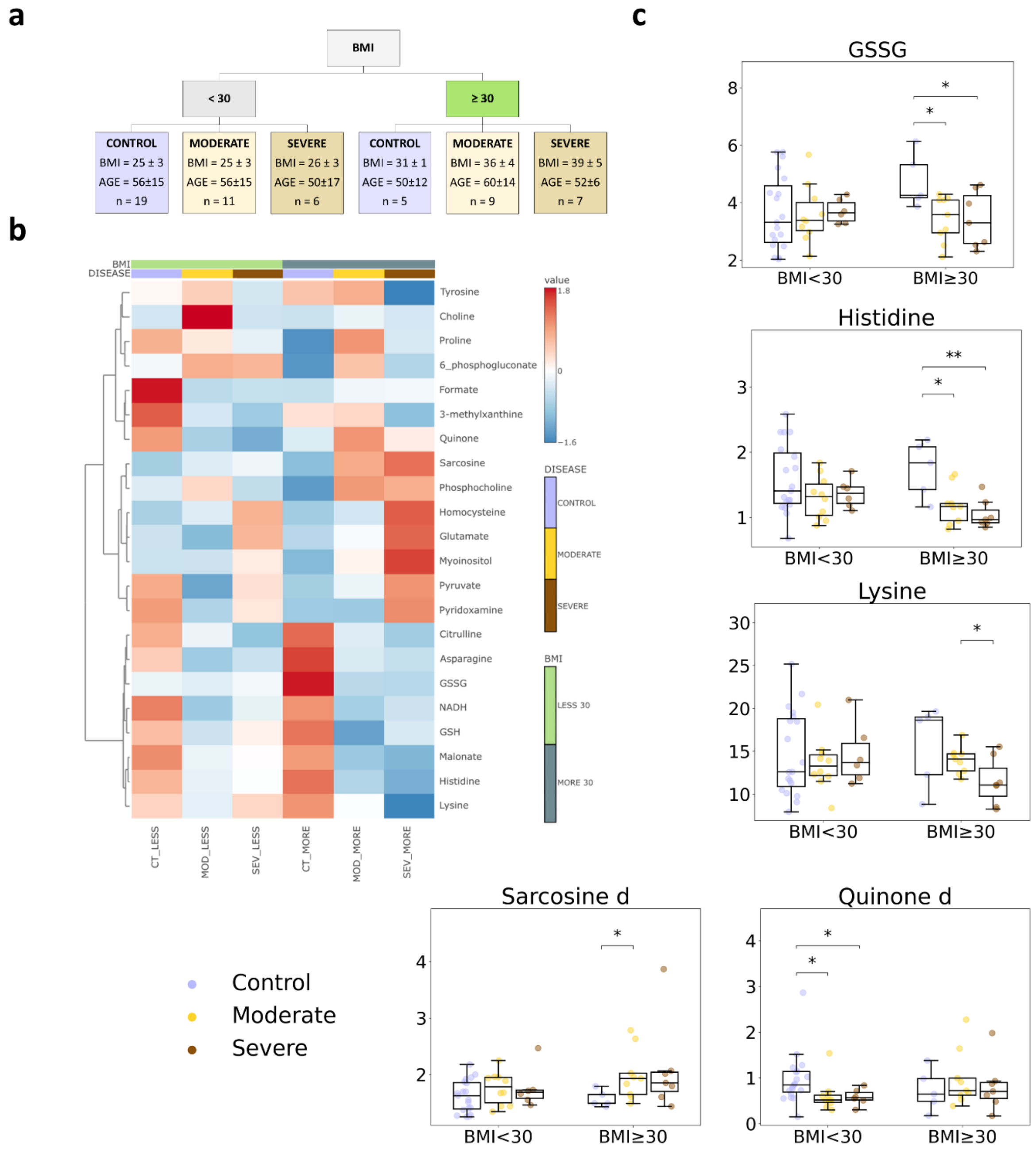

Figure S3. Specific changes in BMI. A) Separation by BMI into less than 30 and equal to or greater than 30 and further division into the three different variables: CONTROL, MODERATE COVID-19 and SEVERE COVID-19. B) Heatmap. C) Boxplot representing the normalized concentrations of metabolites that differ between BMI < 30 and BMI ≥ 30. P-value < 0.05 is indicated by a *, ** indicates a p-value < 0.01. GSSG = oxidized glutathione, GSH = reduced glutathione.

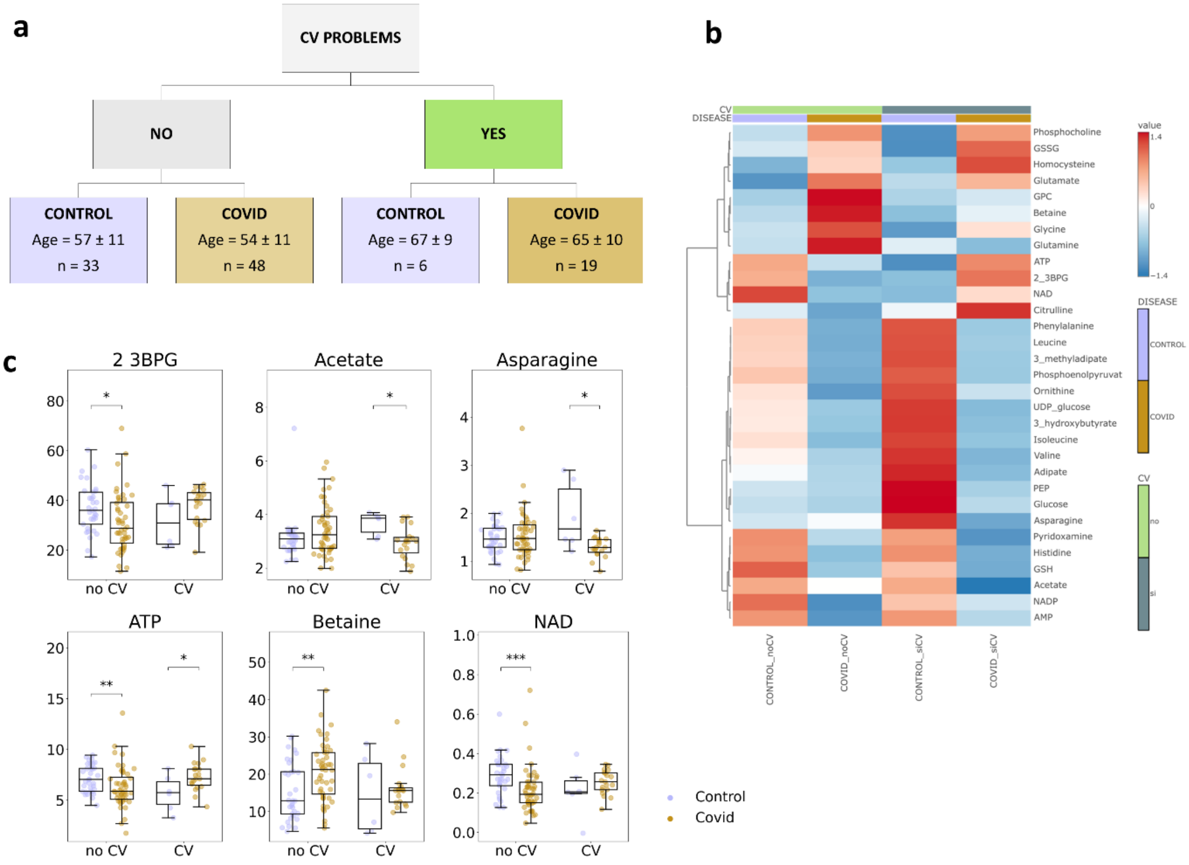

Figure S4. Specific changes in Cardiovascular problems. A) Separation into groups according to whether they suffer cardiovascular problems, and further division into CONTROL patients and COVID patients. B) Heatmap. C) Boxplot representing the normalized concentrations of metabolites that differ between CV and no CV. GSSG = oxidized glutathione. * indicates a p-value < 0.05, ** indicates a p-value < 0.01, *** indicates a p-value < 0.001.

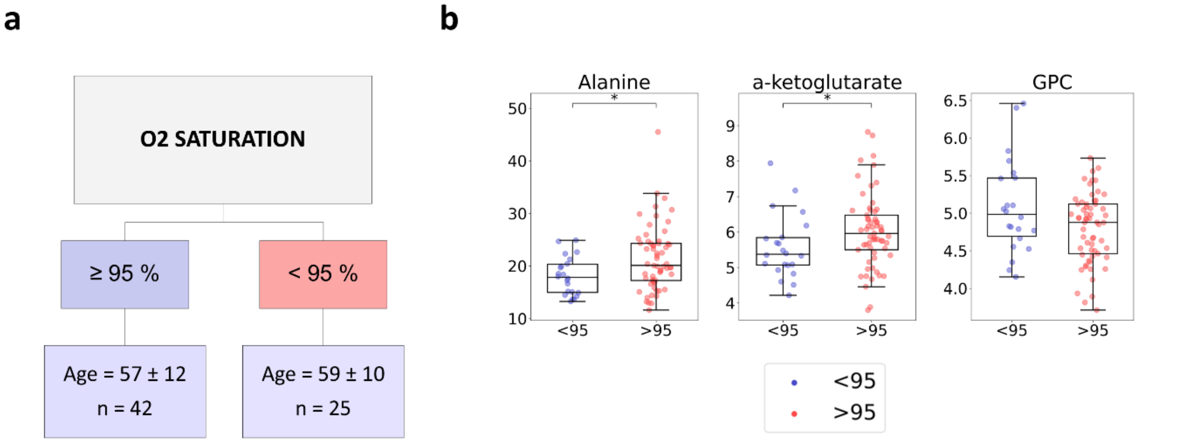

Figure S5. Specific changes in O2 Saturation. A) Separation into groups according to the percentage of O2 saturation in COVID-19 patients. B) Boxplot representing the normalized concentrations of metabolites that differ between O_2 Sat < 95% and O_2 Sat ≥ 95%. P-value < 0.05 is indicated by a *. GSH = reduced glutathione, GPC = glycerophosphocholine.

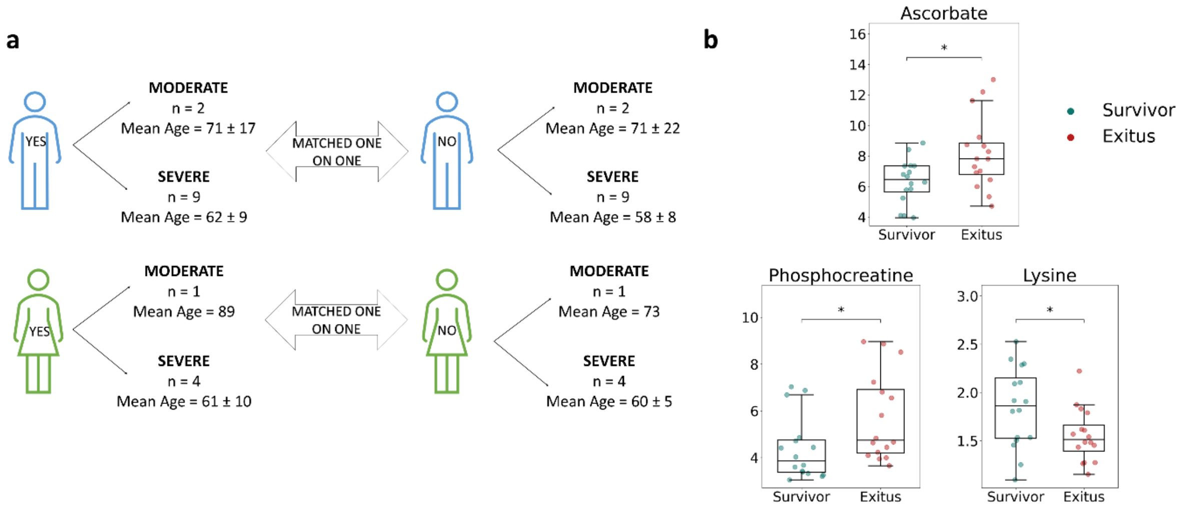

Figure S6. Specific changes in ECMO A) MODERATE COVID-19 and SEVERE COVID-19 patients were individually matched with respect to gender, age, diabetes, and cardiovascular problems. B) Boxplot representing the normalized concentrations of metabolites that differ between ECMO and NO ECMO. P-value < 0.05 is indicated by a *. GSSG = oxidized glutathione, GSH = reduced glutathione.

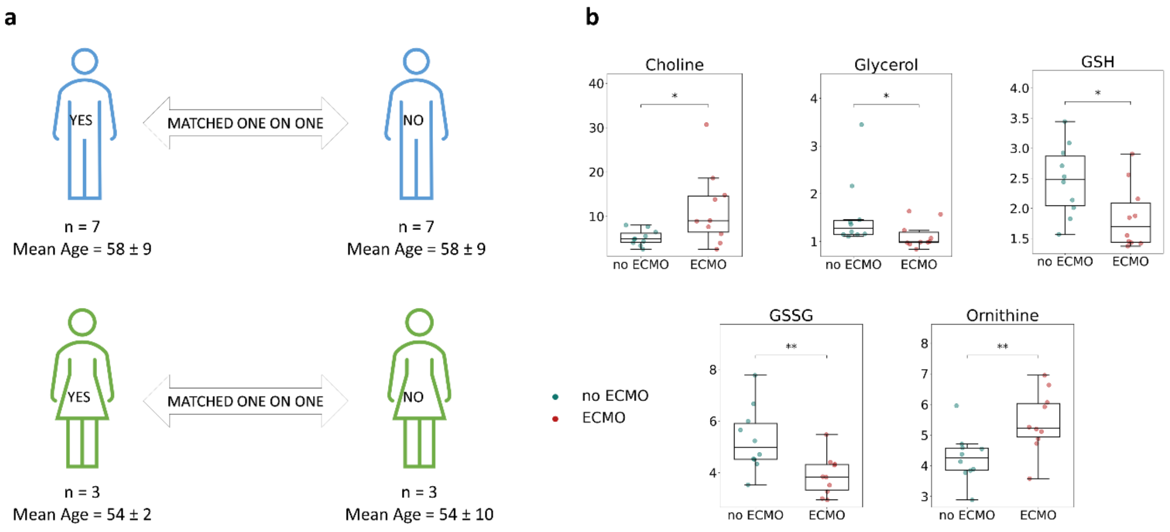

Figure S7. Specific changes in EXITUS. A) SEVERE COVID-19 patients were individually matched with respect to gender, age, diabetes, and cardiovascular problems. B) Boxplot representing the normalized concentrations of metabolites that differ between EXITUS and NO EXITUS. * indicates a p-value < 0.05, ** indicates a p-value < 0.01.

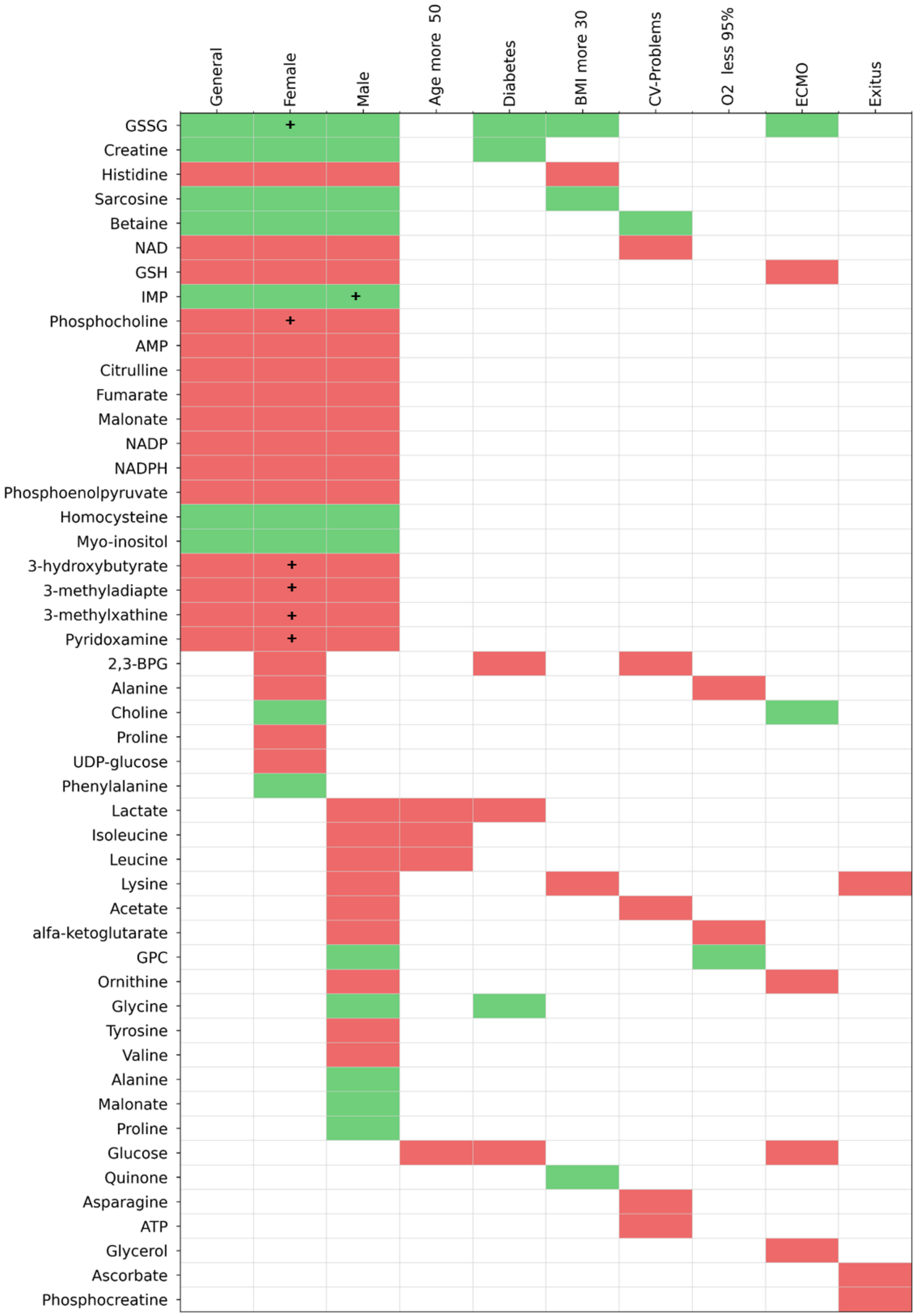

Figure S8. Shematic overview of metabolomics changes in RBCs of COVID-19 patients. Metabolites that increase are labelled in green and metabolites that decrease are labelled in red. 3BPG = 2,3-

bisphosphoglycerate, AMP = adenosine monophosphate, ATP = adenosine triphosphate, GSH = reduced glutathione, GSSG = oxidized glutathione, GPC = glycerophosphocholine, Hcy = homocysteine, IMP = Inosine monophosphate, NAD = Nicotinamide Adenine Dinucleotide, NADP = Nicotinamide Adenine Dinucleotide Phosphate, NADPH = Nicotinamide Adenine Dinucleotide Phosphate, GPC = glycerophosphocholine. Changes labelled with a + are not significant, but have a very clear tendency.
